# Predictive models for suicidal thoughts and behaviors among Spanish University students: rationale and methods of the *UNIVERSAL (University & mental health)* project

**DOI:** 10.1186/s12888-016-0820-y

**Published:** 2016-05-04

**Authors:** Maria Jesús Blasco, Pere Castellví, José Almenara, Carolina Lagares, Miquel Roca, Albert Sesé, José Antonio Piqueras, Victoria Soto-Sanz, Jesús Rodríguez-Marín, Enrique Echeburúa, Andrea Gabilondo, Ana Isabel Cebrià, Andrea Miranda-Mendizábal, Gemma Vilagut, Ronny Bruffaerts, Randy P. Auerbach, Ronald C. Kessler, Jordi Alonso, on behalf of the UNIVERSAL study group.

**Affiliations:** Health Services Research Group, IMIM (Hospital del Mar Medical Research Institute), Barcelona, Spain; Pompeu Fabra University (UPF), Barcelona, Spain; CIBER Epidemiología y Salud Pública (CIBERESP), Madrid, Spain; University of Cadiz (UCA), Cádiz, Spain; Institut Universitari d’Investigació en Ciències de la Salut (IUNICS-IDISPA), University of Balearic Islands (UIB), Palma de Mallorca, Spain; Miguel Hernandez University of Elche (UMH), Alicante, Spain; University of the Basque Country (UPV-EHU), San Sebastian, Spain; Outpatient Mental Health Care Network, Osakidetza-Basque Health Service. Biodonosti Health Research Institute, San Sebastian, Spain; Department of Mental Health, Corporació Sanitaria Parc Taulí, Sabadell, Spain; Universitair Psychiatrisch Centrum, KULeuven (UPC-KUL), Leuven, Belgium; Department of Psychiatry, Harvard Medical School, Boston, MA USA; Center for Depression, Anxiety and Stress Research, McLean Hospital, Boston, MA USA; Department of Health Care Policy, Harvard Medical School, Boston, MA USA; IMIM (Hospital del Mar Medical Research Institute), PRBB Building, Doctor Aiguader 88, 08003 Barcelona, Spain

**Keywords:** Suicide, Mental health, University students, Cohort studies, Risk factors, Protective factors, Predictive modelling

## Abstract

**Background:**

Suicide is a leading cause of death among young people. While suicide prevention is considered a research and intervention priority, longitudinal data is needed to identify risk and protective factors associate with suicidal thoughts and behaviors. Here we describe the *UNIVERSAL* (*University and Mental Health*) project which aims are to: (1) test prevalence and 36-month incidence of suicidal thoughts and behaviors; and (2) identify relevant risk and protective factors associated with the incidence of suicidal thoughts and behaviors among university students in Spain.

**Methods:**

An ongoing multicenter, observational, prospective cohort study of first year university students in 5 Spanish universities. Students will be assessed annually during a 36 month follow-up. The surveys will be administered through an online, secure web-based platform. A clinical reappraisal will be completed among a subsample of respondents. Suicidal thoughts and behaviors will be assess with the Self-Injurious Thoughts and Behaviors Interview (SITBI) and the Columbia-Suicide Severity Rating Scale (C-SSRS). Risk and protective factors will include: mental disorders, measured with the Composite International Diagnostic Interview version 3.0 (CIDI 3.0) and Screening Scales (CIDI-SC), and the Epi-Q Screening Survey (EPI-Q-SS), socio-demographic variables, self-perceived health status, health behaviors, well-being, substance use disorders, service use and treatment. The *UNIVERSAL* project is part of the International College Surveys initiative, which is a core project within the World Mental Health consortium. Lifetime and the 12-month prevalence will be calculated for suicide ideation, plans and attempts. Cumulative incidence of suicidal thoughts and behaviors, and mental disorders will be measured using the actuarial method. Risk and protective factors of suicidal thoughts and behaviors will be analyzed by Cox proportional hazard models.

**Discussion:**

The study will provide valid, innovative and useful data for developing prevention programs for youth suicide and for improving early identification for high-risk students. The longitudinal design of this study will improve causal interpretation of analyzed associations, needed for generating and validating predictive models. It will represent the first results about suicidal thoughts and behaviors in the Spanish university population. The World Mental Health Survey collaboration will permit accurate cross-national comparisons.

## Background

Among individuals aged 15-29 years, suicide is a leading cause of death in many European countries [1]. And, for every person who completes suicide, it is believed that 25 people attempt [1, 2]. Given the enormity of the socioeconomic and emotional burden [3, 4], the European Commission and the World Health Organization (WHO) have identified suicide prevention as a core public health target – with a particular focus on adolescents and young adults [5].

Adolescence and early adulthood is a key developmental period [6]. On the one hand, it is time when youth learn to more effectively navigate stress, hone effective coping strategies, and develop the competencies, attitudes, values, and social network necessary to make a successful transition into adulthood [7, 8]. Conversely, it also is a peak period of mental disorder onset, as it is estimated that 75 % of mental disorders have an age of onset [9]. Moreover, earlier disorder onset is associated with worse prognosis and greater suicide risk [10].

For some, the transition to university may be stressful and increase risk for mental disorder onset [11, 12]. Perhaps one of the most challenging aspects is managing increased psychosocial stress and academic pressures in a new, unfamiliar environment. Presently, in the United States (US), suicide is the second leading cause of death for university students [13, 14] and between 4 and 10 % of college students report having serious suicidal thoughts in the previous 12 months [15]. In Spain, mental health research in university students is limited [16–18], and thus, there is a dire need to better understand suicidality within this important population segment.

Research synthesized by the Centers for Disease Control and Prevention (CDC) suggests that risk and protective factors vary as a function of age, gender, and ethnicity [19]. Although previous research has explored these factors in other geographical contexts (e.g., United States) [20, 21] it is not clear if these findings would extend to Spanish university students. There are some differences in the transition from school to university in other countries. For example, in Spain, in contrast to US, a majority of the students reside at home or close to their family and they are financially supported by their family during the university period. Thus the stress associated with this transition might be weaker than in other countries. On the other hand, in Spain, although universities have their own psychological or counselling services, the amount of services offered is typically lower than in other countries, such as the US. This might imply a lower access to specific services.

To address these critical gaps in our knowledge, we have initiated the *UNIVERSAL* project. Here we present the overall study background, objectives, design, and analysis plan. The project is part of the International College Surveys initiative in the context of the World Mental Health (WMH) surveys consortium (http://www.hcp.med.harvard.edu/wmh/), an international initiative created to improve the scientific knowledge about suicidal behavior among university students worldwide. The general objective of the *UNIVERSAL* project is to assess the prevalence and the incidence of suicidal thoughts and behaviors in Spanish university students. Mental disorders and academic performance are evaluated both as associated factors as well as relevant health and social outcomes. Further, cross-national analyses will be completed to test differences across WMH countries.

The study will address the following issues: i) test prevalence of suicidal thoughts and behaviors among first-year university students in Spain; ii) test incidence of suicidal thoughts and behaviors up to 36 months; iii) identify relevant risk associated with a higher incidence of suicidal thoughts and behaviors, and iv) delineate protective factors associated with less incidence of suicidal thoughts and behaviors.

## Methods

### Study design

As a part of the World Mental Health International College Student (WMH-ICS) project (see http://www.hcp.med.harvard.edu/wmh/college_student_survey.php), the *UNIVERSAL* project is an ongoing multicenter, observational, and prospective cohort study of first-year university students followed over 36 months. Surveys will be conducted via an online, secure web-based platform. Additionally, a clinical reappraisal interview will be completed in a subsample of respondents to ensure validity of assessment methods of mental disorders. Predictive models of suicidal thoughts and behaviors will be estimated and validated. At the time of the manuscript submission the study is in the data collection stage.

### Setting

Five public universities from different regions of Spain participate: Cadiz University (UCA); Balearic Islands University (UIB); Basque Country University (UPV-EHU); Pompeu Fabra University (UPF); and Miguel Hernández University (UMH). These universities represent about 8 % of the undergraduate enrollment capacity annually offered in Spain.

### Eligibility and recruitment

*Inclusion criteria:* a) ages18 to 24 years; and b) enrolled in the first year of university for the first time. *Exclusion criteria:* a) poor knowledge of Spanish language; and b) not accept the informed consent to the study. Students under 18 years old are eligible when they turn 18 and satisfy other inclusion criteria. Based on eligibility criteria we estimate that about 18,000 students will be eligible to participate.

All first year undergraduates from participating universities will be invited to participate. Invitation methods across different college campuses, including advertising campaigns at the campus (e.g., stands with information, information in the classrooms, university web) and up to 4 personal e-mail invitation letters from the university authorities. To increase participation, a raffle of academic material (€ 40) is held at the end of the second year among all respondents who complete the surveys.

### Data collection on-line platform

#### Registration and verification

To participate in the study, eligible students are invited to complete the study registration form first through the *UNIVERSAL* website (see https://encuesta.estudio-universal.net/) and must agree with the informed consent by checking the “*I Agree*” checkbox. In the registration form, students are asked to provide personal contact information, so that they can be re-contacted to enter the survey. A verification e-mail is then automatically sent to the student’s University e-mail address, which includes a personal access code to the baseline survey (web link and personal password) and a copy of the informed consent form.

#### Timing of assessments

**Fig. 1:**
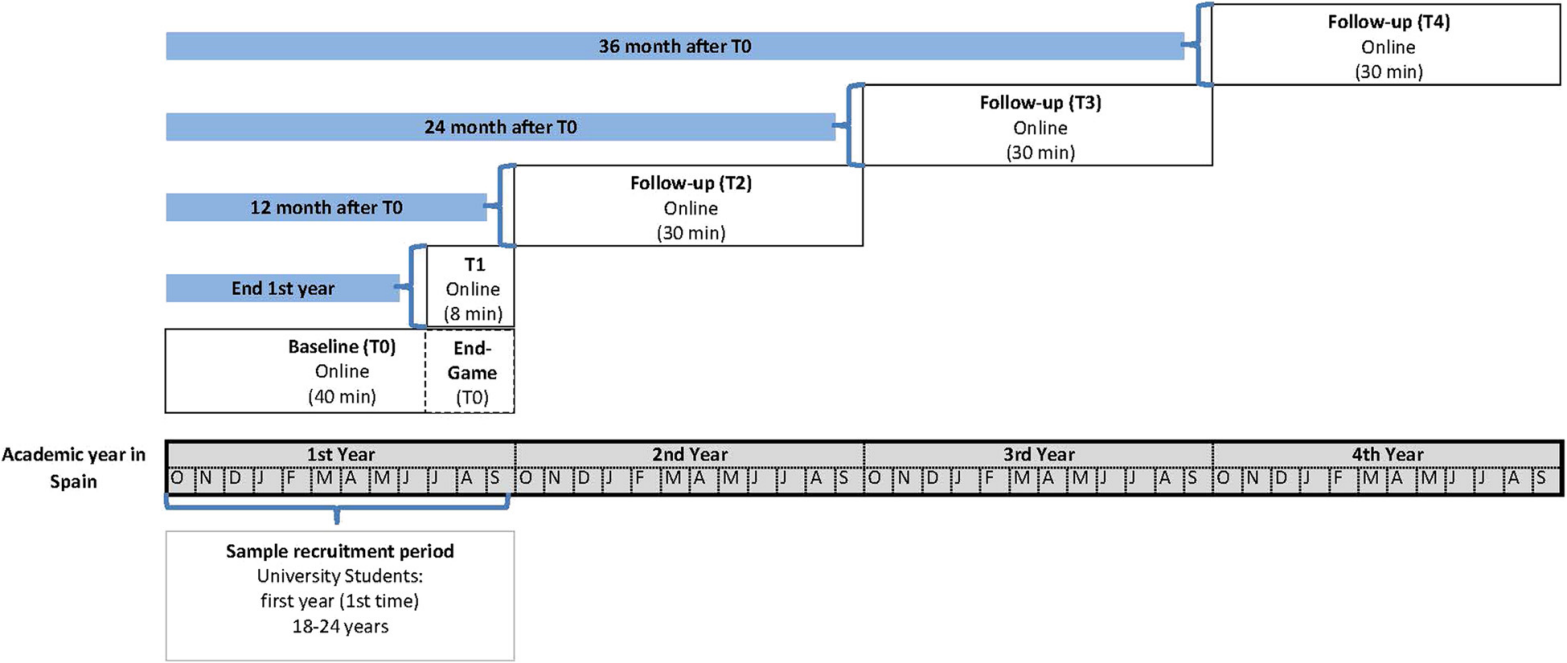
Timeline study design. The *UNIVERSAL (University and Mental Health)* project

Respondents will be complete five assessments throughout the study. The baseline survey (T0) is completed after registration, during the 1st year of the university degree (from October to July). The administration time of T0 is about 40 min. Students who complete at least 5 % of the T0 survey will receive an invitation e-mail at the end of the first year (from July to September) with an electronic link to complete a brief online survey (T1). The T1 survey will include questions pertaining to students’ first year experiences at the university. The information gathered at T1 supplements the baseline data (e.g., academic performance) and the expected administration time is 8 min. Then, 12, 24 and 36 months after completion of the baseline survey (T0), respondents will be invited to complete follow-up questionnaires (T2-T3-T4, respectively), with an estimated administration time of 30 min. The study timeline is depicted in Fig. 1.

#### Data collection platform (DCP)

The DCP is specially designed and developed for the *UNIVERSAL* project, follows international recommendations and guidelines for computerized assessment (International Test Commission–ITC, 2005). This ensures proper use, technical handling, quality control, and security of the data. The system allows for automatic e-mail reminders to complete the survey, storage of responses as well as output reports of participation and data consistency checks throughout the interview, ensuring for instance that the responses provided are within acceptable ranges and checking for consistency across ages reported throughout the interview (e.g., age of onset younger or equal the age at interview). The link to the survey that is sent to each student is personal, associated to a single code, and it is active for a limited period of time. If the participant does not access the DCP within a week, an automatic email reminder is sent. For security reasons, the platform renews the links and sends new access links and passwords to all the respondents who have never accessed the questionnaire or who have not completed the survey.

### Study questionnaire

Based on previous development form vulnerability-stress models [22–25] that distinguish between vulnerability or distal factors, and stressors or proximal factors, our project gathers self-reported data about suicide thoughts and behaviors (ideation, plans, and attempts) and candidate risk and protective factors: sociodemographic, self-perceived health status, health behaviors, mental wellbeing, mental disorders (attention-deficit hyperactivity disorder (ADHD), major depressive episode (MDE), bipolar disorder (BP), generalized anxiety disorder (GAD), panic disorder (PD), posttraumatic stress disorder (PTSD), eating disorders, and psychosis), alcohol and substance use disorders, history of suicidal thoughts and behaviors, and non-suicidal self-injuries (NSSI), services use and treatment, personal history, stressful live events, psychological factors (personality, hopelessness, stress management, impulsivity and anxiety coping), sexuality, religiosity/spirituality, university expectations and experiences, and academic performance.

The questionnaire content is summarized in Table 1 and is described in more detail below.

**Table 1:** Study variables, model factors and assessment timing. The UNIVERSAL (University and Mental Health) project

Section	Subsections/Variables		Vulnerability	Stress	Protective	T0	T1	T2-T4	Original instrument
	Suicidal related behaviors (lifetime and 12 month)								
		Death wishes		*		+	+	+	
		Ideation		*		+	+	+	SITBI
		Plan		*		+	+	+	C-SSRS
		Attempt		*		+	+	+	
		Non-suicidal self-injury		*		+	+	+	
Section 1)	Socio-demographic data								
		Age				+		+	
		Gender	*			+			
		Nationality		*		+			
		Race				+			
		Grade		*		+			
		Student status (part time/full time)				+		+	
Section 2)	Self-perceived health status								
		Psysical and Mental General Health		*	*	+	+	+	CIDI 3.0; CIDI-SC
		Disability		*		+		+	SDS
		Chronic conditions		*		+		+	
		Injuries and trauma		*		+		+	Army STARRS
Section 3)	Healthy behaviors								
		Activity			*	+		+	YRBS
		Diet							CIDI 3.0
		Sleep		*		+		+	
Section 4)	Well-being								
		Well-being			*	+		+	WEMWBS
Section 5)	Mental disorders (lifetime and 12 month)					+		+	
		ADHD		*		+	+	+	ASRS
		Major depressive episode		*		+	+	+	
		Bipolar disorder		*					CIDI 3.0; CIDI-SC
		Panic disorder		*		+	+	+	EPI-Q SS
		Generalized anxiety disorder		*		+		+	
		Eating disorders		*		+		+	CIDI-SC
		PTHD		*		+		+	
		Psychotic disorders		*		+		+	
Section 6)	Subtance use disorders								
		Alcohol abuse		*		+	+	+	AUDIT-10
		Other substancies		*		+	+	+	ASSIST
Section 7)	Internet behavior							+	IBQ
Section 8)	Service use and treatment								
		Health services and treatment use			*	+	+	+	LCS; J-MHAT 7; ARMY; CIDI 3.0; SCS; Katrina; LCS
Section 9)	Personal history								
		Psychiatric family history	*			+			CIDI 3.0
		Adverse childhood experiences	*			+			ACES; CTQ
		Family support	*		*	+			ARMY; PSSMS; SF-TAI; ARMY
		Previos school bullying	*			+			BS; J-MHAT 7
Section 10)	Stressful life events								LEQ; 2009 YRBS,
		Stressful Life Events within 12 month		*		+	+	+	HRB-ADMP; ARMY; CIDI 3.0
									DRRI
Section 11)	Psicological factors								
		Personality traits	*	*	*	+			TIPI
		Hopelessness		*		+		+	BHS
		Stress Management			*	+			SESE
		Impulsivity		*		+		+	UPPS
		Anxiety coping			*	+			ARMY; Katrina
Section 12)	Sexuality			*		+		+	(Williams Institute, 2009)†
Section 13)	Religiosity/spirituality				*	+			CIDI 3.0
Section 14)	University								
		University expectations				+			SDPS; PTWHA
		University experiencies		*	*		+	+	SDPS
		Academic performance		*			+	+	

* Type of factor; + Factor included in questionnaire; † Best Practices for Asking Questions about Sexual Orientation on Surveys (The Williams Institute, 2009)

*Abbreviations*: ACES = Adverse Childhood Experiences Scale; ADHD = Attention-deficit/hyperactivity disorder; ASRS = Adult ADHD Self-Report Scale; ARMY = ARMY STARRS; ASSIST = Alcohol Substance Involvement Screening Test; AUDIT-10 = The Alcohol Use Disorders Identification Test; BHS = Beck Hopelessness Survey; BS = The Bully Survey; CIDI 3.0= Composite International Diagnostic Interview version 3.0; CIDI-SC = Composite International Diagnostic Interview Screening Scales; C-SSRS = Columbia-Suicide Severity Rating Scale; CTQ = Childhood Trauma Questionnaire; DRRI = Deployment Risk and Resilience Inventory; EPI-Q SS = EPI-Q Screening Survey; HRB-ADMP = Department of Defense Survey of Health Related Behaviors among Active Duty Military Personnel; IBQ = Internet Behavior and Addiction; J-MHAT = Joint Mental Health Advisory Team 7; IHP =  Internalized Homophobia scale; Katrina = Katrina Survey – 12 month Follow-up Version; LCS = Land Combat Study; LEQ = Life Events Questionnaire; MDS = Multiple Discrimination Scale; PSSMS = Psychological Sense of School Membership Scale; PTWHA = Part-Time Work and Hurried Adolescence: The Links among Work Intensity, Social Activities, Health Behaviors, and Substance Use; SCS = States of Change Scale; SDPS = Seattle Development Project Survey – School Risk and Protective Factors; SDS = Shehaan Disability Scale; SESE = Student Experience and Student Expectations; SF-TAI = Short Form Test Anxiety Inventory; SITBI = Self-Injurious Thoughts and Behaviors Interview; TIPI = Ten Item Personality Inventory; 2009; UPPS = Impulsive Behavior Scale; WEMWBS = Warwick-Edinburgh Mental Well-being scale; YRBS = Youth Risk Behavior Survey

#### Suicidal related behaviors

Suicidal thoughts and behaviors items are taken mostly from the Self-Injurious Thoughts and Behaviors Interview (SITBI) [26] and Columbia-Suicide Severity Rating Scale (C-SSRS) [27]. Items probe death wishes (“*wish you were dead or would go to sleep and never wake up?”*), suicidal ideation *(“have thoughts of killing yourself?”*), suicide plans (“*think about how you might kill yourself [e.g. taking pills, shooting yourself] or work out a plan of how to kill yourself?”*), and suicide attempts *(“make a suicide attempt [i.e., purposefully hurt yourself with at least some intent to die]?”*). Several questions about Non-suicidal self-injury (NSSI) query *(hurting yourself on purpose, without wanting to die? [for example, cutting yourself, hitting yourself or burning yourself]?).* All items will be assessed about the past 12 months and lifetime.

#### Mental disorders

Attention deficit hyperactivity disorder (ADHD), major depressive episode (MDE), bipolar disorder (BP), generalized anxiety disorder (GAD), panic disorder (PD), posttraumatic stress disorder (PTSD), eating disorders, and psychosis will be assessed. Item prompts are drawn from versions of the Composite International Diagnosis Interview version 3.0 (CIDI 3.0) [28], and Screening Scales (CIDI-SC) [29], and Epi-Q Screening Survey (EPI-Q-SS) [30]. Adult ADHD Self-Report Scale (ASRS) [31] will be used to assess attention deficit hyperactivity disorder (ADHD). Evaluation of depressive episode (MDE), bipolar disorder (BD), generalized anxiety disorder (GAD), and panic disorder (PD) include items about onset of the disorder and presence during the last 12 month. Posttraumatic stress disorder (PTSD), eating disorders and psychosis will be assessed with adapted screening items from CIDI-SC.

#### Sociodemographic factors

Age, gender, nationality, race and descriptive information about their status at the university (e.g. grade, part time/full time) will be collected.

#### Self-perceived health status

Perceptions of physical and mental health “overall physical/mental health” will be evaluated with items adapted from CIDI Screening Scales (CIDI-SC). Additionally, disability and medical conditions (e.g., chronic health problems, head injury) will be evaluated. Disability will be measured with selected items adapted from Sheehan Disability Scale (SDS) [32].

#### Health behaviors

Health behaviors (e.g., exercise, sleep) diet will be evaluated with items from the Youth Risk Behavior Survey (YRBS) [33] and CIDI 3.0.

#### Mental well-being

The short version of Warwick-Edinburgh Mental Well-Being Scale instrument (SWEMWBS) [34], which had been validated for the Spanish general population [35] and university students [36], will be utilized.

#### Substance Use (alcohol and substances)

The Alcohol Use Disorders Identification Test, 10-item version (AUDIT-10) [37], the Alcohol Substance Involvement Screening Test (ASSIST) [38] and selected items from the CIDI 3.0. will be used to screen substance use problems and disorders.

#### Services use and treatment

A selection of items from the Land Combat Study (LCS) [39], Joint Mental Health Advisory Team 7 (J-MHAT 7) [40], Stages of Change Readiness and Treatment Eagerness Scale SOCRATES [41], Katrina Survey [42], and CIDI 3.0 will be used to assess: (a) current and past use of psychological counseling and medication as well as (b) barriers to and motivation for seeking treatment.

#### Personal history

Psychiatric family history, adverse childhood experiences, and family support will be assessed with parts of the CIDI 3.0. Adverse Childhood Experiences Scale (CES) [43], and Childhood Trauma Questionnaire (CTQ) [44]. Bullying experiences and other interpersonal violence are collected with the Bully Survey (BS)[45] and J-MHAT 7 [40]. Additional childhood information includes questions about childhood experience, perceived social support and social network at school-age.

#### Stressful life events

Stressful experiences in the past 12 month will be collected with set of items from Live Events Questionnaire (LEQ) [44], Deployment Risk and Resilience inventory [46] and others developed for department of defense survey of health related behaviors among active duty military personnel [47] and for Army STARRS [29]. Items include: (a) injury and/or death of friends or family members, (b) romantic break-ups, (c) life-threatening events, (d) financial stressors, (e) health stressors, (f) social stressors.

#### Psychological factors

We include: personality, hopelessness, impulsivity and anxiety coping/stress management. Personality traits will be evaluated with Ten Item Personality Inventory (TIPI) [48], which probes extraversion, agreeableness, conscientiousness, emotional stability and openness to experience. Hopelessness will be assessed with selected items from Beck Hopelessness Scale (BHS) [49]. Four items from UPPS Impulsive Behavior scale (UPPS) [50] will be used for evaluate impulsive behavior.

#### Sexuality

Sexuality items were developed based on recommendations from best practices [51]. Sexuality items will probe sexual orientation, sexual behavior, and discrimination related to sexual orientation. Multiple Discrimination Scale (MDS) [52] and Internalized Homophobia scale (IHP) [53] will be administrated for students who answer non-heterosexual orientation.

#### Religiosity/spirituality

Preferences and level of involvement in religious/spirituality events reflect items from the CIDI 3.0.

#### University expectations and experiences

Expectations about alcohol or drug consumption at university will be collected with items from the Seattle Social Development Project Survey [54]. Other items in this section probe: (a) student experience and expectations in university [55], (b) reasons for attending university, (c) where they are living. In the follow-up surveys, a set of questions about involvement in activities during the university year, satisfaction with university services, and intentions to leave universities studies are probed.

#### Academic performance

Final grades will be tracked among students in the follow- up surveys and objectively calculated by information from Universities administration offices.

### Adaptation of the questionnaires into Spanish

Many instruments used in this study already have a Spanish version available and have been used in several previous studies. Examples include the SITBI [56], the CIDI 3.0 [57] and AUDIT [58]. Adaptations of the remaining instruments and items into Spanish were performed by the project study group (approximately 15 % of the questionnaire content). A forward translation by two independent teams was carried out. Each independent team included at least one linguist professional and one mental health professional. An expert panel took part in the adaptation, identifying, and resolving discrepancies between the forward translations. Further adaptations into Basque and Catalan languages (using the Spanish version as the source) were performed in the same way for universities with more than one official language (e.g., Basque for UPV-EHU and Catalan for UPF, UIB, and UMH).

### Study quality control procedures

Data quality control procedures will be implemented and the results will be reported on a weekly basis with regard to the following aspects: survey participation and duration, and individual and item level quality data.

#### Survey participation

A table with the number of students registered to the survey and summary of the participation, and completion rate. The table includes a summary of the validation status of registered individuals as provided by the universities. Approximately once a month the corresponding universities will validate new registrations and will report the causes of exclusion. For each participant, there will be three possible validation status: valid (i.e., inclusion criteria are fulfilled), excluded (i.e., inclusion criteria are not fulfilled), or pending validation.

#### Survey duration

A table will report descriptive statistics of the duration time for each University and the overall sample. Additionally, the number of individuals below and over a pre-specified threshold will be reported in order to identify possible outliers. We have set as values to flag attention about response quality: a total time of less than 15 min. and of more than 60 min (as they correspond to the 25^th^ and 75^th^ percentiles of the response distribution).

#### Quality checks at the individual level

To identify possible unreliable respondents, the respondents with a higher proportion of empty responses and a higher proportion of “*I don’t want to answer”* responses will be listed and the corresponding proportions will be displayed.

#### Quality checks at the item level

In order to identify problematic items or possible skip errors of the interview, those items with a higher proportion of empty responses and those not willing to answer are listed. The 95^th^ percentile cutoff will be used to identify candidate problematic items.

More details on the data quality control procedures are provided in Table 2.

**Table 2:** Quality control indicators. The *UNIVERSAL (University and Mental Health)* project

Indicator	Operational indicator	Action required
*Participation and completion Report:*		
*- Registered respondents:* Individuals registered to the study	*n* (% over eligible)	
*- Not started surveys:* Respondents that have not accessed the link to the survey, among registered		
*- Incomplete surveys:* Respondents that have entered the survey but have not completed it, among registered	*n* (%)	
*- Completed surveys:* Respondents that have finalized the whole interview, among registered	*n* (%)	
Validation status:		
*- Valid respondents:* Registered respondents that fulfill eligibility criteria	*n* (%)	
*- Excluded respondents:* Registered respondents that do not satisfy eligibility	*n* (%)	
*- Validation Pending:* Registered respondents pending to be validated	*n* (%)	
Data quality indicators:		
*- Survey duration table:* Descriptive information on duration time by university and overall.	Mean (SD^a^) Median P25^b^ P75^c^ min. max.	
*- Assessment of problematic respondents based on time:* students with survey duration time below and over specific thresholds^d^	*n* (%)	➔ The respondents with duration below and above the threshold are listed
*• Assessment of problematic respondents based on item completeness:* Surveys with number of items left unanswered or with response “I do not want to answer” over percentile 95	P95^e^	➔ The respondents with number of items left unanswered and with higher number of responses *“I do not want to answer”* over Percentile 95 are listed. The proportion of items left unanswered for each respondent is evaluated to determine acceptability of the survey.
*• Assessment of problematic items:* In order to identify problematic items or possible skip errors of the on-line questionnaire, those items with a higher proportion of empty responses and those not willing to answer are listed.	P95^e^	➔ The items with high proportion of incompleteness (if any) are evaluated in terms of understandability, sensitive question level or possible skip errors.

^a^Standard deviation, ^b^Percentile 25, ^c^Percentile 75, ^d^that are considered short and large enough based on the number of items administered to all respondents, ^e^Percentile 95

### Confidentiality and ethical issues

Information collected will be treated as strictly confidential, in compliance with the provisions of the Spanish Law 41/2002, 14^th^ November, and the 1999 Spanish Data Protection Act (LOPD). The *UNIVERSAL* project was approved by Parc de Salut MAR-Clinical Research Ethics Committee. Reference number 2013/5252/I. All the information will be protected in compliance with data gathering procedures following the Code of Ethics and the Helsinki Declaration (Seoul 2008 revision).

An online informed consent is obtained for every respondent. The respondent must agree with the informed consent by checking the “*I Agree*” checkbox in https://encuesta.estudio-universal.net/es/inscripcion.

#### Participant alerts

Although inquiring about suicidal behavior does not increase the risk of suicidal behavior, it is important to minimize any possible risk [59, 60]. Hence, at the end of the survey, all respondents will receive a general notification which provides information on how to access specialized mental health services. For the participants responding “yes” to any of the screening items for suicidal thoughts and behavior (i.e., ideation, plan or intent) or NSSI in the last 12 month, a specific alert will provide information for consulting with a health professional.

### Clinical reappraisal

The instrument used in the *UNIVERSAL* project for assessing mental disorders is an adapted version of the CIDI 3.0 and CIDI-SC for which a good concordance with clinical diagnoses has been reported in both instruments [61, 62, 63]. However given that the instrument includes additional items from other scales, as well as the fact that it has not been tested among university students so far, it was decided to perform a clinical reappraisal.

The Mini International Neuropsychiatric Interview 5.0.0 (MINI) [64] will be administrated to a subsample of respondents shortly after the completion of the online survey, to assess diagnostic concordance between. Administration of the MINI will be completed by trained mental health professionals by telephone. The following MINI sections are administered; Major Depression Episode (MDE); Hypomania/Mania (BP); Panic disorder (PD); Generalized Anxiety Disorder (GAD); Substance Use Disorders (SUD). Also, the Suicidal Behavior is reappraised. For consistency with the survey data, the MINI has been adapted as necessary to account for lifetime and 12-month recall periods, with the exception of SUD, for which only 12 months are assessed. Interviewers will be blinded to participants’ mental health status. Eligible respondents will be selected after completion of T0 and after completion of T2.

### Statistical power calculations

The statistical power needed for the main result has been computed for a universe estimated of 18,000 eligible subjects (undergraduate students enrolled for the first time). The absolute precision that would be achieved for the prevalence estimate of suicidal thoughts and behaviors, assuming a 12-month prevalence of 10 % (conservative estimate) and a significance level alpha of 0.05 has been evaluated for three different assumptions of baseline response rate: a) for a response rate of 60 % (*n* = 10,800) precision would be ±0.40 %; b) for a response rate of 30 % (*n* = 5,400) precision would be equal to ±0.57 %; and c) for a response rate of 15 % (*n* = 2,700) precision would be ±0.81 %.

**Fig. 2:**
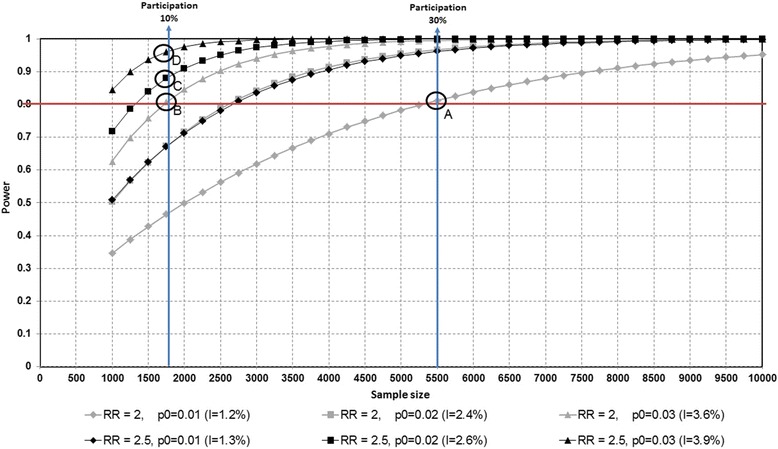
Statistical power for different study sample sizes. The *UNIVERSAL (University and Mental Health)* project. Statistical Power* to detect relative risk of various sizes (relative risk of 2 or 2.5) versus study sample size*Power is obtained assuming significance level alpha of 0.05 (unilateral contrast), a fixed prevalence of exposure to a given risk factor of 20%, and different levels of probability of the event in the non-exposed group (p0=0.01, p0=0.02 or p0=0.03). Acronyms: RR= Relative risk, I= Incidence, p0= probability of the event among the non-exposed.Points highlighted in the graph: RR = 2 and p0 = 0.01 (point A); RR=2 and p0 = 0.03 (point B); RR = 2.5 and p0 = 0.02 (point C); and RR = 2.5 and p0 = 0.03 (point D)

With regard to the relative risk (RR), different scenarios are presented graphically (see Fig. 2), showing how power increases with sample size (i.e., final participation rate at follow-up), for significance level alpha of 0.05, using a unilateral contrast and assuming a fixed prevalence of exposure to a given risk factor of 20 %, two different levels of relative risk to be detected (with Ho: RR < =1), RR = 2 (in gray) or RR = 2.5 (in black), and three different levels of the event probability in the non-exposed group (p0) : p0 = 0.01 (diamond), p0 = 0.02 (square), p0 = 0.03 (triangle). In a restrictive scenario where we want to detect a RR = 2 assuming p0 = 0.01 (i.e., overall low incidence rate of 1.2 %) (see point A in the figure), a final sample size of *n* = 5,500 students (equivalent to a final participation of 30 %) would be needed to achieve a statistical power over 80 %. In the case scenario of a final sample size of *n* = 1,750 (i.e. final participation rate of almost 10 %) we would reach a power = 0.81 for a RR = 2 and p0 = 0.03, representing an overall incidence rate of 3.6 % (see point B in the figure). For RR = 2.5, a power of 0.88 would be reached if p0 = 0.02 (point C) and a power of 0.96 if p0 = 0.03 (point D). The test statistic used was the pooled variance Z-test.

For the clinical reappraisal study, a random sample of 100 subjects with a mental disorder and 100 subjects without a mental disorder will be selected. A sample of 100 from the positive group and 100 from the negative group would achieve 80 % power to detect a difference of 0.10 between the area under the ROC curve (AUC) under the null hypothesis of 0.80 and an AUC under the alternative hypothesis of 0.90, using a one-sided z-test at a significance level alpha of 0.05. The data are discrete (rating scale) responses. The AUC is computed between false positive rates of 0.10 and 0.15

## Analysis plan

### Survey response and participation bias assessment

Study representativeness depends on the extent to which participation is high and respondents are representative of the target population. Participation rates are continuously monitored the calculation of response rate) using the American Association for Public Opinion Research (AAPOR) definitions [65]. In case of a low response rate (i.e., below 50 %), a two-phase sampling for non-response [66], denominated “end-game strategy”, will be carried out. The end-game strategy consists of selecting a random subsample of eligible students that have not been enrolled in the study so far from each university and offer them an economic incentive (range € 5 to 25) to complete the interview. End-game respondents could be considered an approximation to non-responders and will be compared to initial respondents in terms of a selection of variables of interest. Weights will be applied in the analysis and response rate calculations to restore the probabilities of selection for the end-game strategy [65].

#### Participation bias assessment

A key issue in survey research is whether respondents differ from non-respondents in some way that is likely to impact systematically on the estimated outcome values. To evaluate possible bias, the distribution of the sample in terms of age, sex, and university degree categories, among others, will be compared with aggregated information provided by the participating universities. If differences are found, post-stratification weights will be used to restore distributions population distributions in each University in terms of these variables.

### Prevalence, incidence and risk and protective factors: modeling

At T0, lifetime prevalence and prevalence in the last 12-months will be estimated for suicidal ideation, plans, and attempts. Additionally, the prevalence of the mental disorders assessed will be calculated. Cumulative incidence of suicidal thoughts and behavior and mental disorders will be measured at follow-up using the actuarial method.

The effect of risk and protective factors on suicidal thoughts and behaviors will be evaluated through Cox proportional hazards models. Candidate variables to be included in the models are the variables indicated in the Table 1. Different models will be estimated for suicidal ideation, plans and attempts. Cox proportional hazards models will be used to derive separate risk equations of developing suicidal thoughts and suicidal behavior within a real time in the future, among first year university students. Discrimination ability (the ability of the model to separate individuals who develop the event from those who do not) will be assessed with the C statistic. Model calibration, to assess how accurately model predictions match overall observed event rates, will be evaluated with a version of the goodness of fit of Hosmer and Lemeshow developed by D’Agostino and Nam [67]. Internal model validation will be carried out with k-fold cross-validation methods. The external validity of the models will be assessed using data from similar studies conducted in other countries (i.e., Belgium, United States, among others) within the WMH surveys initiative.

In all future publications we will follow the STROBE criteria (Strengthening the Reporting of Observational Studies in Epidemiology) [68]

### Clinical calibration

Diagnostic accuracy of the scales of mental disorder included in the questionnaire will be carried out with ROC curves analysis, including the area under the ROC curve (AUC) and the estimation of diagnostic performance indices for different cut off points (i.e., sensitivity, specificity, positive predictive value, negative predictive value, positive and negative likelihood ratios and diagnostic odds ratio).

## Discussion

### Strengths

Presently, there is limited information on protective factors of suicidal behaviors among university students, and the current study sought to provide valid, innovative and useful data for developing prevention programs for youth suicide and for improving early identification for high-risk students.

The longitudinal design of this study will enable us to develop predictive models, and importantly, this project will provide the first data on suicidal thoughts and behaviors among Spanish university students -- with data from different regions in the country. It is important to emphasize that cross-national data will permit the comparison with other countries of the WMH Surveys initiative. This will increase the magnitude of observations and will offer relevant information about differences between countries. Finally, the online platform provides an efficient data collection methodology, which is especially useful in young populations. There is evidence that it conveys more reliable information about suicidal behavior [69].

### Limitations

Although the validity of the instruments used to derive the final content of the survey questionnaire is well-established, additional information will be necessary to establish the validity: i) of the modifications introduced in the questionnaire; and ii) in this particular population group. It is for this reason that we will perform the clinical reappraisal study. Its results will be used to calibrate prevalence data as well as to conduct sensitivity analyses of the predictive models of risk factors.

A major challenge for this study is the potential low participation and/or the loss of subjects during the follow-up. Low response/retention rates would reduce the statistical power and can generate a selection bias which affect at the generalization of the results. The potential low participation will be minimized with successive invitation to participate reminders and with an end-game strategy. In this strategy, non-responders will be randomly selected for a specific. An important effort for reducing the loss during the follow-up it will be also necessary.

Finally it should be noted that although there is geographical variation in the universities participating in the *UNIVERSAL* project, its external validity will be still limited, as they are part of a convenience rather than random sampling strategy. Some results of this study will not be extrapolated to students from other universities or to non-university students. However, incidence and predictive models will not be affected by this bias.

### Potential impact

The *UNIVERSAL* project had the potential to provide valid, new, and useful knowledge about important risk and protective factors. The project will emphasize the development and validation of predictive information based on these factors. To the extent that factors are modifiable, the results of this project have the potential to inform and improve future intervention strategies.

Interventions are more effective when there is concurrent focus on both risk and protective factors [70]. The *UNIVERSAL* project has the potential to provide useful predictive models based on both types of factors, and thus, will inform early identification, prevention, and treatment.

Finally, as the study will be replicated in several countries around the world, as part of the WMH International College Surveys initiative, international comparisons will be made possible. They will inform about universal factors as well as cultural and context factors that are associated with suicidal behaviors among university students.

### Ethics approval and consent to participate

The UNIVERSAL project was approved by the Parc de Salut MAR-Clinical Research Ethics Committee (*Reference number 2013/5252/I*). All the information will be protected in compliance with data gathering procedures following the Code of Ethics and the Helsinki Declaration (Seoul 2008 revision).

An online informed consent is obtained for every respondent. The respondent must agree with the informed consent by checking the “*I Agree*” checkbox in https://encuesta.estudio-universal.net/es/inscripcion. The authorization to check with the universities if they are first year students from the participating universities is obtained too through the consent informed page. The informed consent include: i) Information regarding the research project; ii) Details about confidentiality, and data management and storage; iii) project contact for more information]. The respondent testify: i) I have read the information regarding the research project; ii) I have received enough information about the study; iii) I was able to request additional information about the study; iv) I understand that my participation is voluntary and can withdraw from the study; v) I hereby agree to participate in the study; and vi) I have read the information concerning measures to ensure confidentiality and protection of my data. A copy of the informed form is sent to the respondent by mail.

### Consent to publish

Non Applicable.

### Availability of data and materials

Data: Non applicable.

Materials: Can be obtained through the correspondent author (email address: jalonso@imim.es)

## Abbreviations

AAPOR: public opinion research
ADHD: attention deficit hyperactivity disorder
ASRS: adult ADHD self-report scale
ASSIST: alcohol substance involvement screening test
AUC: area under the ROC curve
AUDIT: alcohol use disorders identification test
BHS: beck hopelessness scale
BP: bipolar disorder
BS: bully survey
CDC: centers for disease control and prevention
CES: adverse childhood experiences scale
CIDI 3.0: composite international diagnosis interview
CIDI-SC: composite international diagnostic interview screening scales
C-SSRS: Columbia-suicide severity rating scale
CTQ: childhood trauma questionnaire
DCP: survey data collection platform
EPI-Q-SS: epi-Q screening survey
GAD: generalized anxiety disorder
IHP: internalized homophobia scale
J-MHAT 7: joint mental health advisory team 7
LCS: land combat study
LEQ: live events questionnaire
MDE: major depressive episode
MDS: multiple discrimination scale
MINI: mini international neuropsychiatric interview
NSSI: non-suicidal self-injury
PD: panic disorder
PTSD: posttraumatic stress disorder
RR: relative risk
SDS: Sheehan disability scale
SITBI: self-injurious thoughts and behaviors interview
SOCRATES: stages of change readiness and treatment eagerness scale
STROBE: strengthening the reporting of observational studies in epidemiology
SUD: substance use disorders
SWEMWBS: Short Warwick-Edinburgh Mental Well-being Scale
TIPI: ten item personality inventory
UCA: University of Cadiz
UIB: University of Balearic Islands
UMH: Miguel Hernandez University
UPF: Pompeu Fabra University
UPPS: UPPS impulsive behavior scale
UPV-EHU: University of the Basque Country
WHO: world health organization
WMH: world mental health
YRBS: youth risk behavior survey
